# Basal Cell Carcinoma Arising in a Previous Full-Thickness Graft Donor Site: A Case Report and Comprehensive Literature Review

**DOI:** 10.3390/jcm14020591

**Published:** 2025-01-17

**Authors:** Amanda Y. Shen, Ishith Seth, Gianluca Marcaccini, Warren M. Rozen, Richard J. Ross

**Affiliations:** 1 Department of Plastic and Reconstructive Surgery, Peninsula Health, Melbourne, VIC 3199, Australia; amanda.yang.shen@gmail.com (A.Y.S.);; 2 Faculty of Medicine and Surgery, Monash University, Melbourne, VIC 3004, Australia

**Keywords:** basal cell carcinoma, BCC, full-thickness graft, graft, tumor latency

## Abstract

**Background/Objectives:** Basal cell carcinoma (BCC), the most common skin malignancy, typically occurs in sun-exposed areas but can develop in atypical locations, such as scars, burns, and skin graft donor sites. BCC arising specifically in full-thickness skin graft donor sites is exceptionally rare. This study presents a unique case of BCC occurring 16 years post-graft harvesting and provides a comprehensive literature review to analyze clinical patterns, possible etiopathogenesis, and treatment strategies. **Methods**: A case report was described and a comprehensive literature review was conducted using PubMed, Scopus, and Web of Science (up to November 2024). Studies were screened for cases of BCC involving skin graft donor and recipient sites. Extracted data included demographics, graft type, latency period, histopathology, treatment, and outcomes. **Results**: A 68-year-old woman presented with biopsy-confirmed mixed nodular and micronodular BCC at the donor site of a full-thickness skin graft 16 years after its use for nasal reconstruction. Surgical excision with clear margins resulted in complete resolution without recurrence. A literature analysis revealed seven cases of graft-associated BCC, predominantly affecting older females. Partial-thickness grafts were frequently involved, with latency periods ranging from 1 to 61 years. Nodular BCC was the most common histological subtype, and surgical excision remained the primary and most effective treatment. **Conclusions**: Although rare, BCC can develop in skin graft donor sites after prolonged latency. Chronic trauma, impaired vascularization, and genetic alterations likely contribute to tumorigenesis. Lifelong surveillance, early detection, and timely intervention are critical to improving outcomes.

## 1. Introduction

Basal cell carcinoma (BCC) is the most common malignant skin tumour, accounting for approximately 80% of non-melanoma skin cancers globally, particularly in Caucasian populations [1]. The development of BCC is primarily associated with cumulative ultraviolet (UV) light exposure, with lesions predominantly occurring on sun-exposed areas such as the head and neck [2]. However, BCC can also arise in atypical locations, including non-photo-exposed regions such as scars, burns, vaccination sites, tattoos, areas of chronic inflammation, and skin grafts [3,4]. These uncommon presentations suggest a multifactorial etiology involving both environmental and genetic factors, such as trauma-induced carcinogenesis and field cancerization effects [4,5].

Skin grafts, frequently utilized for reconstructive purposes following burns, trauma, and oncologic surgeries, provide functional and esthetic restoration. Despite their clinical benefits, grafted regions and donor sites may become susceptible to secondary pathological changes, including neoplasms such as BCC [4]. BCC development in graft-associated regions is particularly concerning due to altered tissue microenvironments, including chronic inflammation, reduced vascularization, and impaired immune surveillance, which collectively create conditions conducive to carcinogenesis [6,7,8]. These regions are also subjected to mechanical stress and trauma, which may promote DNA damage and subsequent tumorigenesis through mechanisms similar to those observed in Marjolin’s ulcers [9]. Previous studies have reported BCC developing on skin grafts, with latency periods ranging from 1 year to 61 years post-surgery [6]. In these cases, nodular and invasive BCC subtypes have been the most observed histological patterns, with partial-thickness grafts being the most frequently implicated [5,6]. Full-thickness grafts, though less commonly associated with BCC, may exhibit unique histopathological features due to their distinct dermal–epidermal architecture. Additionally, donor site location, particularly in non-sun-exposed regions, appears to influence the subtype, with superficial BCC being more common in areas shielded from UV radiation [10]. These observations underscore the need for long-term dermatologic surveillance, particularly in patients with extensive grafting histories.

The mechanisms underlying BCC development in grafted or donor skin still need to be better understood. While UV exposure remains a critical factor, other contributing mechanisms include reduced vascularization, decreased elasticity, and chronic trauma to grafted or donor areas, which may predispose the overlying epithelium to carcinogenesis [4,7]. Furthermore, genetic mutations in the *PTCH1* and *SMO* genes, key components of the Hedgehog signalling pathway, have been implicated in BCC’s pathogenesis, with up to 90% of sporadic cases demonstrating these alterations [8]. Such genetic changes may drive tumorigenesis even in non-photo-exposed regions, emphasizing the role of intrinsic factors in BCC development [7,8].

While numerous cases of BCC arising in graft recipient sites have been reported, its occurrence in full-thickness skin graft donor sites remains exceedingly rare. Most documented cases involve partial-thickness grafts, with very few reports focusing on full-thickness grafts [5,6,10]. Given the unique properties of full-thickness grafts, which include the transfer of the entire epidermis and dermis, distinct pathophysiological mechanisms may influence the risk of neoplasm development in these areas.

This study presents a rare case of BCC arising in a previous full-thickness skin graft donor site, accompanied by a comprehensive literature review. This report aims to underscore the clinical course, possible etiopathogenesis, and management strategies for BCC in graft-associated regions. Additionally, we emphasize the importance of long-term surveillance in patients undergoing skin graft procedures, as this case highlights an uncommon but significant post-reconstructive complication.

## 2. Materials and Methods

### 2.1. Study Design

This study is a case report and comprehensive literature review. It was performed according to the principles of the Declaration of Helsinki for medical research involving human subjects. The patient gave written informed consent to publish the case details and associated clinical images.

### 2.2. Literature Search Methodology

A comprehensive literature search was conducted across three major electronic databases: PubMed, Web of Science, and Scopus. The search spanned from their respective inceptions to November 2024. The search strategy incorporated a combination of Medical Subject Heading (MeSH) terms and free-text keywords, using Boolean operators (“AND”/“OR”) to ensure a thorough search. The keywords included “Basal cell carcinoma” OR “BCC”, “Skin graft” OR “Full-thickness graft” OR “Split-thickness graft”, “Donor site” OR “Recipient site”, “Cancer seeding” OR “Tumour inoculation”, and “Recurrence” OR “Secondary malignancy”. Filters were applied to include studies published in English and involving human subjects. Additional articles were identified by manually screening reference lists from retrieved publications to capture potential studies.

### 2.3. Data Extraction

Data extraction was performed systematically, and relevant information from all eligible studies was organised into a table format. Extracted data included:

1. Study details: Author names and year of publication.
2. Patient demographics: Age and sex of patients.
3. Graft characteristics: Donor site, recipient site, and graft type (full-thickness or split-thickness).
4. Clinical timeline: Latency period (time from surgery to BCC onset).
5. Histological subtype: Specific histopathological findings of BCC.
6. Management and outcomes: Treatment methods and recurrence data where available.

## 3. Case Presentation

A 68-year-old woman presented to the clinic with a biopsy-confirmed nodular basal cell carcinoma on the right upper neck, located within the scar of a previous full-thickness skin graft donor site. The patient reported a history of nodular BCC of the nasal dorsum 16 years prior, which was treated with surgical excision and reconstruction using a full-thickness skin graft harvested from the current site of the lesion. The initial procedure was uneventful at another hospital, and the nasal graft site has remained disease-free since. The patient denied any history of significant sun exposure, sunburns, or previous skin cancers outside the documented lesion. Her past medical history was notable due to a multinodular goitre and polymyalgia rheumatica, although she was not receiving immunosuppressive therapy. She was a non-smoker and had been retired for several years, working primarily in an office-based occupation with limited UV exposure.

**Figure 1:**
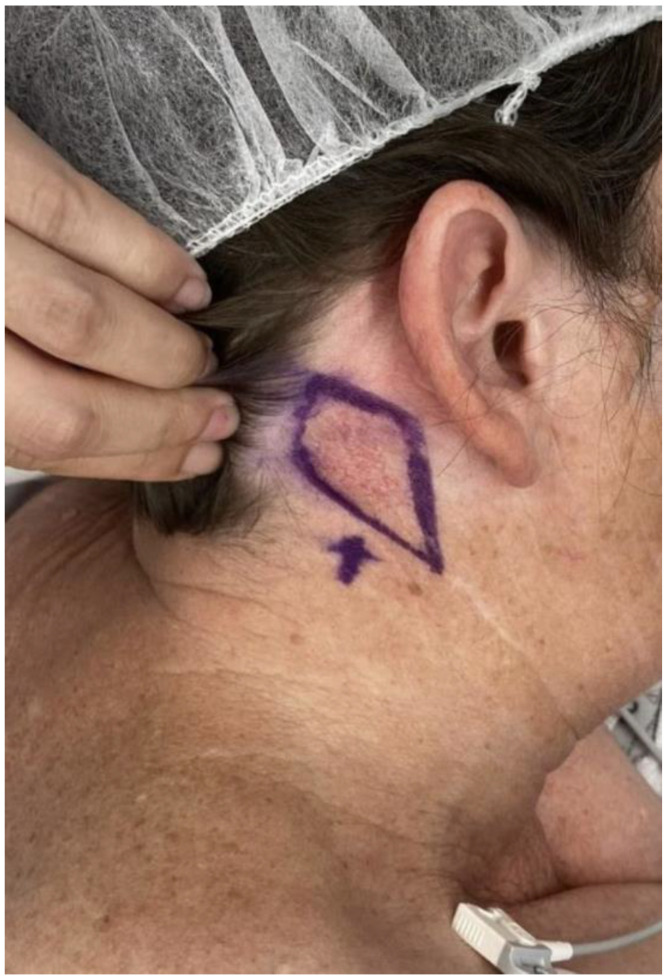
Pre-operative clinical photograph of the basal cell carcinoma arising in an old graft donor site scar.

On physical examination, a slightly raised telangiectatic lesion measuring approximately 3 cm in diameter was observed at the centre of the scar from the previous graft donor site on the right neck (Figure 1). The lesion appeared well-demarcated and erythematous, consistent with BCC’s clinical features. Additionally, the nasal dorsum displayed a well-healed, full-thickness skin graft with no evidence of tumor recurrence, ulceration, or abnormal pigmentation.

The patient underwent surgical excision of the lesion with direct closure of the defect. Histopathological examination revealed a mixed nodular and micronodular BCC, confirming the diagnosis. The tumor was noted to have clear lateral and deep margins. Attempts to retrieve additional details regarding the prior operation, including procedural notes and the surgical instruments used, were unsuccessful due to the closure of the facility where the initial surgery had been performed several years ago. This case highlights the rare occurrence of BCC arising at a full-thickness skin graft donor site over a prolonged latency period. It underscores the need for long-term donor and recipient graft site follow-ups in patients undergoing reconstructive surgery for cutaneous malignancies.

## 4. Results

The findings from this review, summarized in Table 1, highlight key clinical patterns of basal cell carcinoma arising in skin graft donor and recipient sites. Among the seven cases analyzed, most patients were female (5/7), aged 50–80 years, predominantly in older individuals. The thigh was the most frequent donor site (five cases), while recipient sites varied, including the presternal area, forearm, scalp, upper eyelid, and mandibular angle. Partial-thickness grafts were the most commonly used graft (six cases), with only two cases involving full-thickness grafts. The latency period ranged widely, from 1 year to 61 years, with an average exceeding 20 years [3,11]. Histologically, nodular BCC was the most prevalent subtype (four cases), followed by superficial and invasive BCC. A unique case reported by Imbernón-Moya et al. involved the simultaneous onset of superficial multifocal BCC at both donor and recipient sites [2]. Management in all cases involved surgical excision, with adjunct therapies such as imiquimod cream and photodynamic therapy being applied in select cases. No recurrences were reported during follow-up, underscoring favourable outcomes with appropriate treatment and highlighting the need for long-term surveillance of grafted regions.

Further analysis revealed distinct histopathological patterns in cases involving full-thickness grafts compared to partial-thickness grafts, with a higher proportion of nodular and micronodular subtypes in the former. Notably, latency periods were longer in cases involving full-thickness grafts, averaging 31 years compared to 16 years for partial-thickness grafts. This observation may reflect the more robust dermal–epidermal architecture transferred during full-thickness grafting, potentially influencing delayed tumorigenesis. In addition, donor sites located in regions with reduced UV exposure demonstrated a higher likelihood of superficial BCC subtypes, suggesting a contributory role of site-specific factors in determining BCC histopathology. Among the cases reviewed, one patient experienced a transient adverse effect from photodynamic therapy, including localized erythema and mild discomfort, which resolved within 48 h. While all cases demonstrated favourable outcomes post-surgical excision, the need for adjunctive therapies in cases of incomplete excision underscores the importance of achieving clear margins. These findings collectively underscore the significance of individualized surveillance protocols, particularly in older patients or those with extensive graft histories.

## 5. Discussion

Basal cell carcinoma is the most common form of non-melanoma skin cancer; its development is primarily associated with cumulative ultraviolet radiation exposure, with lesions frequently arising on sun-exposed regions such as the head and neck [1,2]. However, BCC’s occurrence in atypical, non-photo-exposed sites, such as scars, burns, chronic inflammatory regions, and skin graft donor or recipient sites, highlights the multifactorial nature of BCC pathogenesis [3,4]. This rare case report describes an unusual case of BCC developing in a full-thickness skin graft donor site 16 years after its use in nasal reconstruction. This finding contributes to the limited literature on secondary malignancies arising in graft-associated regions, emphasizing the clinical significance of long-term surveillance and exploring potential etiopathogenic mechanisms.

While UV exposure remains a primary driver of BCC, its development in non-photo-exposed sites such as graft donor regions necessitates the exploration of additional factors. Chronic trauma, inflammation, and impaired vascularization create a microenvironment conducive to neoplastic transformation [4,5]. Previous studies have reported similar processes in Marjolin’s ulcers, where chronic inflammation leads to malignant changes over time [6]. These mechanisms are particularly relevant in graft donor sites, where tissue integrity, elasticity, and immune surveillance are significantly altered due to graft harvesting [5,7].

Field cancerization, defined as widespread epithelial damage resulting in a precancerous state, is another proposed mechanism for BCC development in graft sites [4]. Imbernón-Moya et al. documented a unique case of simultaneous BCC development in both donor and recipient sites, suggesting that cumulative genetic alterations predisposed the epithelial tissue to synchronous tumorigenesis [2]. This phenomenon likely involves mutations in key components of the Hedgehog signalling pathway, such as *PTCH1* and *SMO*, present in up to 90% of sporadic BCC cases [8]. These mutations may occur de novo or due to trauma or impaired repair mechanisms following graft harvesting.

Additionally, immunologic dysregulation plays a significant role in skin carcinogenesis, particularly in immunocompromised populations like renal transplant recipients (RTRs) [9]. RTRs exhibit a higher incidence of NMSC due to prolonged immunosuppressive therapy, with calcineurin inhibitors and azathioprine exacerbating the carcinogenic effects of UV exposure [10,13]. Matinfar et al. reported a pooled incidence of 12.6% for NMSC among RTRs, with BCC accounting for 2.2% of cases [13]. Although our patient was immunocompetent, this association highlights the heightened vulnerability of grafted or altered skin to malignancies when protective mechanisms are compromised.

The latency period for BCC development in graft-associated regions varies widely, ranging from 1 year to 61 years, as documented in prior studies [5,6]. Yazici et al. reported the longest latency period of 61 years, underscoring the prolonged nature of tumorigenesis in non-photo-exposed areas [3]. In our case, the latency period was 16 years, aligning with previously reported averages. This variability emphasizes the importance of lifelong surveillance for patients with a history of skin graft procedures, mainly when grafts are harvested for oncologic reconstructions.

Histologically, nodular BCC remains the most prevalent subtype, followed by superficial and invasive variants [4,5]. Our patient presented with mixed nodular and micronodular BCC, which aligns with findings from other studies and highlights the potential for more aggressive subtypes in altered or grafted tissue [2]. Subtle clinical presentations of BCC, particularly in scarred or grafted regions, can complicate early diagnosis, necessitating a high index of suspicion during follow-up evaluations.

The potential for malignant cell seeding during graft harvesting or reconstruction is essential in oncologic surgery. Although rare, tumor inoculation has been documented in melanoma and squamous cell carcinoma, particularly following biopsies or improper handling of surgical instruments [3,14]. Østergaard et al. reported tumor seeding in adjacent tissue following an incisional biopsy of a conjunctival BCC, illustrating the risks of accidental cell transfer [15]. While no evidence supports direct tumor seeding in BCC cases involving donor sites, procedural vigilance remains critical.

In our case, the absence of procedural records precluded confirmation of surgical techniques, such as blade sterility or tissue handling, during the initial operation. Standard oncologic practices, including new blades for graft harvesting and excision of malignant lesions, are essential to minimize the risk of contamination. This principle has been underscored in other reports, such as Angelos et al., who documented a nodular BCC in a scalp graft site [4]. Hypothetically, improper handling of grafted tissue during harvest or transplantation could introduce malignant cells or trigger microtrauma-induced genetic alterations, predisposing the site to later carcinogenesis. Additionally, surgical stress and tissue ischaemia at the donor site may impair immune surveillance, allowing subclinical neoplastic clones to increase over time, particularly in regions with altered vascularization and elasticity.

Management of BCC arising in graft-associated regions follows established treatment protocols for BCC elsewhere, with surgical excision being the gold standard. All cases in the reviewed literature were treated with surgical excision, achieving clear margins and favourable outcomes without recurrence [2,3,4,5,6,7]. Adjunct therapies, such as topical imiquimod and photodynamic therapy, have been employed in select cases to address superficial BCCs or complex donor site lesions [2,7]. In our case, the lesion was successfully excised with no evidence of recurrence, reinforcing the efficacy of timely surgical intervention. Given the prolonged latency periods observed in graft-associated BCCs, long-term follow-up processes remain imperative. Patients with prior graft procedures, particularly those performed for oncologic reconstructions, should undergo regular dermatologic surveillance to facilitate early detection of suspicious changes.

This case highlights basal cell carcinoma’s rare but significant occurrence in a full-thickness skin graft donor site, contributing to the limited literature on BCC in graft-associated regions. While UV exposure remains a dominant etiological factor, the pathogenesis in non-photo-exposed sites involves a complex interplay of chronic trauma, impaired tissue integrity, and genetic mutations within the Hedgehog signalling pathway [8]. The prolonged latency period, observed across multiple studies, underscores the importance of lifelong clinical vigilance for donor and recipient graft sites. Mutations in the *PTCH1* gene, a key component of this pathway, can lead to aberrant activation of a Smoothened protein and subsequent downstream signalling, driving uncontrolled cellular proliferation and tumorigenesis. Hedgehog pathway inhibitors, such as vismodegib and sonidegib, have been developed to target this mechanism by inhibiting SMO, thereby halting the pathway’s oncogenic effects. The study by Gambini et al. further elucidates the immunomodulatory role of HHIs, demonstrating their ability to disrupt BCC’s immunosuppressive tumor microenvironment [16]. Treatment with HHIs has been shown to enhance adaptive immune responses, increasing cytotoxic T-cell infiltration and upregulating MHC class I expression while also modulating cytokine and chemokine activity. These findings underscore the dual role of HHIs as both targeted molecular agents and facilitators of anti-tumor immune responses, providing a compelling rationale for their use in advanced or inoperable BCC cases. The prolonged latency period, observed across multiple studies, underscores the importance of lifelong clinical vigilance for donor and recipient graft sites. This vigilance is particularly critical, as emerging evidence suggests that therapies targeting the Hedgehog pathway may also influence long-term immune surveillance and treatment resistance mechanisms [15,16,17,18].

The prolonged latency periods of graft-associated BCCs, spanning years to decades, highlight the critical need for lifelong surveillance of both donor and recipient sites [19,20,21,22]. Regular dermatological evaluations utilizing dermoscopy can facilitate early detection of subtle or atypical lesions [23,24,25,26,27]. Artificial intelligence-assisted imaging analyses may enhance early diagnosis by identifying morphological changes indicative of malignant transformation [19]. Patient education is essential for promoting long-term vigilance. Individuals with a history of oncologic skin grafting should be counseled to recognize early signs of BCC, including non-healing ulcers, telangiectasia, and pearly nodules [20]. Given BCC’s multifactorial etiology in graft-associated areas, research into preventative strategies is crucial [26]. Prophylactic interventions, such as topical nicotinamide or systemic chemoprevention, may offer significant benefits, particularly for patients with additional risk factors like immunosuppression or multiple prior skin cancers [28,29,30,31].

While addressing a rare and under-reported phenomenon, this study has several limitations. The primary limitation is the rarity of basal cell carcinoma in full-thickness skin graft donor sites, which restricts the sample size and hampers the generalizability of findings. Most cases in the literature involve partial-thickness grafts, leaving the unique pathophysiological mechanisms of full-thickness graft-associated BCC underexplored. Additionally, the inability to retrieve detailed surgical records for the index case precludes a thorough analysis of intraoperative factors such as blade sterility, graft handling, or other procedural aspects that may contribute to tumorigenesis. The retrospective nature of the literature review also introduces inherent biases, including selection and reporting biases. Many studies reviewed lack consistent follow-up data, making assessing long-term outcomes and recurrence rates challenging. Variability in reporting histopathological details and management strategies further complicates the ability to draw definitive conclusions. Another limitation is the absence of a standardized approach to surveillance protocols across the reviewed cases. The study underscores the importance of lifelong follow-up processes but does not provide robust evidence to define optimal surveillance intervals or modalities. Moreover, while the report speculates on etiopathogenic factors, such as trauma, vascular changes, and genetic alterations, these hypotheses remain unvalidated due to limited data. Lastly, although emerging technologies like artificial intelligence and molecular diagnostics are proposed as future strategies, their practical applicability in this context requires further investigation. Addressing these limitations through prospective multicenter studies and controlled trials will be crucial to refining understanding and improving clinical management of graft-associated BCC.

This report emphasizes the need for strict adherence to oncologic principles during skin cancer surgery, including meticulous surgical techniques to minimize the risk of cell seeding [31]. Further research is warranted to elucidate the pathophysiologic mechanisms underlying BCC development in graft-associated regions and to optimise preventive strategies. A multidisciplinary approach, combining patient education, long-term surveillance, and appropriate management, is essential to improving outcomes for this rare but clinically significant entity [32].

## 6. Conclusions

This case highlights the rare occurrence of basal cell carcinoma arising in a full-thickness skin graft donor site, emphasizing the importance of long-term surveillance for both donor and recipient regions. The prolonged latency period and multifactorial pathogenesis, involving trauma, impaired tissue integrity, and genetic alterations, underscore the need for vigilance in patients with a history of skin grafting. Early detection through routine dermatological assessments, advanced imaging, patient education, and preventative strategies is essential. Emerging technologies, such as artificial intelligence-assisted imaging and predictive analytics, may offer novel tools for earlier detection and risk stratification in such cases. Prophylactic interventions, including topical agents like nicotinamide or immune-modulating therapies, could mitigate the risk of secondary malignancies. Additionally, the role of systemic factors, including inflammation, ischemia-induced hypoxia, and impaired-wound-healing dynamics in graft-associated carcinogenesis, warrants further exploration. Surveillance protocols tailored to patient-specific risk factors, such as graft type, anatomical location, and latency duration, could significantly improve clinical outcomes. Incorporating molecular diagnostics to identify early oncogenic mutations in high-risk graft regions may further enhance early intervention strategies. This report underscores the critical need for multidisciplinary approaches integrating dermatology, oncology, and reconstructive surgery expertise to advance patient care in this complex, under-reported clinical scenario. Further research into the molecular mechanisms and prophylactic interventions for graft-associated BCCs will enhance clinical outcomes and inform future management.

## Figures and Tables

**Table 1:** Summary of reported cases of basal cell carcinoma arising in skin graft donor and recipient sites.

Study	Patient Demographics	Graft Characteristics	Clinical Timeline	Histological Subtype	Management and Outcomes
Cox, 1984 [12]	Female, 80 years	Donor: Thigh (Split-thickness). Recipient: Presternal area	4 years	Superficial	Surgical excision; no recurrence reported
Martin et al., 2005 [6]	Female, 72 years	Donor: Forearm (Split-thickness). Recipient: Forearm	2 years	Nodular, Invasive	Surgical excision; no recurrence reported
Karri et al., 2005 [5]	Female, 52 years	Donor: Left thigh (Split-thickness). Recipient: Right thigh	30 years	Type not specified	Surgical excision; no data on recurrence
Lemierre et al., 2007 [11]	Female, 73 years	Donor: Left inguinal fold (Split-thickness). Recipient: Mandibular angle	1 year	Nodular	Surgical excision; no recurrence reported
Yazici et al., 2011 [3]	Female, 75 years	Donor: Thigh (Full thickness). Recipient: Upper eyelid	61 years	Nodular	Surgical excision; no recurrence reported
Angelos et al., 2013 [4]	Male, 50 years	Donor: Lateral thigh (Split-thickness). Recipient: Scalp	31 years	Invasive	Surgical excision; no recurrence reported
Imbernón-Moya et al., 2015 [2]	Female, 58 years	Donor: Right thigh (Full thickness). Recipient: Right arm	3 years (simultaneous onset)	Superficial, Multifocal	Donor site: Imiquimod and photodynamic therapy, excision; no recurrence reported

## Data Availability

Data supporting reported results can be found in the study.
